# Addressing health corruption during a public health crisis through anticipatory governance: Lessons from the COVID-19 pandemic

**DOI:** 10.3389/fpubh.2022.952979

**Published:** 2022-07-29

**Authors:** Alejandro Gonzalez-Aquines, Iwona Kowalska-Bobko

**Affiliations:** ^1^Institute of Public Health, Faculty of Health Science, Jagiellonian University, Krakow, Poland; ^2^School of Nursing and Healthcare Leadership, Faculty of Health Studies, University of Bradford, Bradford, United Kingdom

**Keywords:** corruption, health systems, anticipatory governance, COVID-19, public health crisis

## Abstract

Corruption in the health sector costs over 500 billion USD annually, weakening health system preparedness and response to health crises like the COVID-19 pandemic. The lack of resources to deal with a shock limits the capacity to protect the population, exposing them to a greater risk of infection and mortality. There is an urgent need to improve health policy to reduce corruption in the health sector during times of crisis. This article aims to propose a prepare and response strategy to address corruption during times of health crises. We first explore the inherent characteristics of health systems that make them vulnerable to corruption and present the different faces corrupt practices take. We then explain why anticipatory governance is fundamental in addressing corruption in health systems and draw upon examples of corruption during COVID-19. Finally, we conclude by proposing that anticipatory governance could decrease the impact of corruption during health crises by increasing the availability of resources required to improve the population's health.

## Introduction

Corruption is the abuse of entrusted power for private gain (1). In the health sector, corruption is considered an ignored pandemic, and it is estimated that over 500 USD billion are lost yearly due to corruption (2). During a health crisis, like the COVID-19 pandemic, the health sector is expected to cope with the shock and protect its population; consequently, countries increase the spending on specific goods or services to reduce the impact of the crisis. For instance, in the early stages of the COVID-19 pandemic, there was an increase in the acquisition of ventilators for severely ill patients and personal protection equipment (PPE) for healthcare personnel. However, due to the nature of a crisis requiring rapid response, countries amend their procurement processes to faster acquisition of equipment, like in the United Kingdom (UK), where laws were approved to create a “VIP” list of providers without the need to go through a tender process (3), Unfortunately, the well-intentioned proposal of reducing procurement times has led to negative consequences as it decreases transparency, harnesses public trust, and hinders countries from responding efficiently to a health crisis.

While it is difficult to calculate the impact of corruption on health outcomes during a public health crisis, the lack of appropriate preparedness and response will undoubtedly affect the capacity of health systems to protect the population's health. A recent study by Farzanegan and Hofmann showed in a cross-country analysis of over 90 countries that an interquartile increase in the corruption index by the World Development Indicators (WDI) reduces the percentage of the population vaccinated for COVID-19 by around 15% (4). The reason behind the difference in vaccination rates relies on the damaging effect corruption has on the capacity of governments to implement health programs efficiently due to the inappropriate use of resources.

The COVID-19 pandemic has shown how corruption comes alongside public health crises. Therefore, the article proposes a prepare and response strategy to address corruption during times of health crises. We first describe why health systems are vulnerable to corruption and present the multiple faces of corruption. We conclude by proposing how anticipatory governance could reduce corruption in times of crisis.

## Methodology

This report is based on a review of the literature conducted in three scientific databases (Scopus, Medline *via* Ovid, and IBSS *via* ProQuest) and gray literature (Google, Transparency International, European Commission) using a combination of “corruption” and similar terms (e.g., fraud, bribery, extortion) and COVID-19 or similar terminology (e.g., SARS-CoV-2, coronavirus). Health system experts from Western and Central-Eastern European countries were also consulted to gather their thoughts on corruption during the COVID-19 pandemic. Moreover, key corruption indicators (e.g., Corruption Perception Index, Rule of Law) were consulted and triangulated with the information collected from the review and interviews with health system experts. The review's findings (not yet published) highlighted the need to comprehend the innate vulnerability of health systems, the multiple faces of corruption in the health sector, and the priority of implementing anticipatory governance for future public health crises.

## The vulnerability of health systems

**Figure 1:**
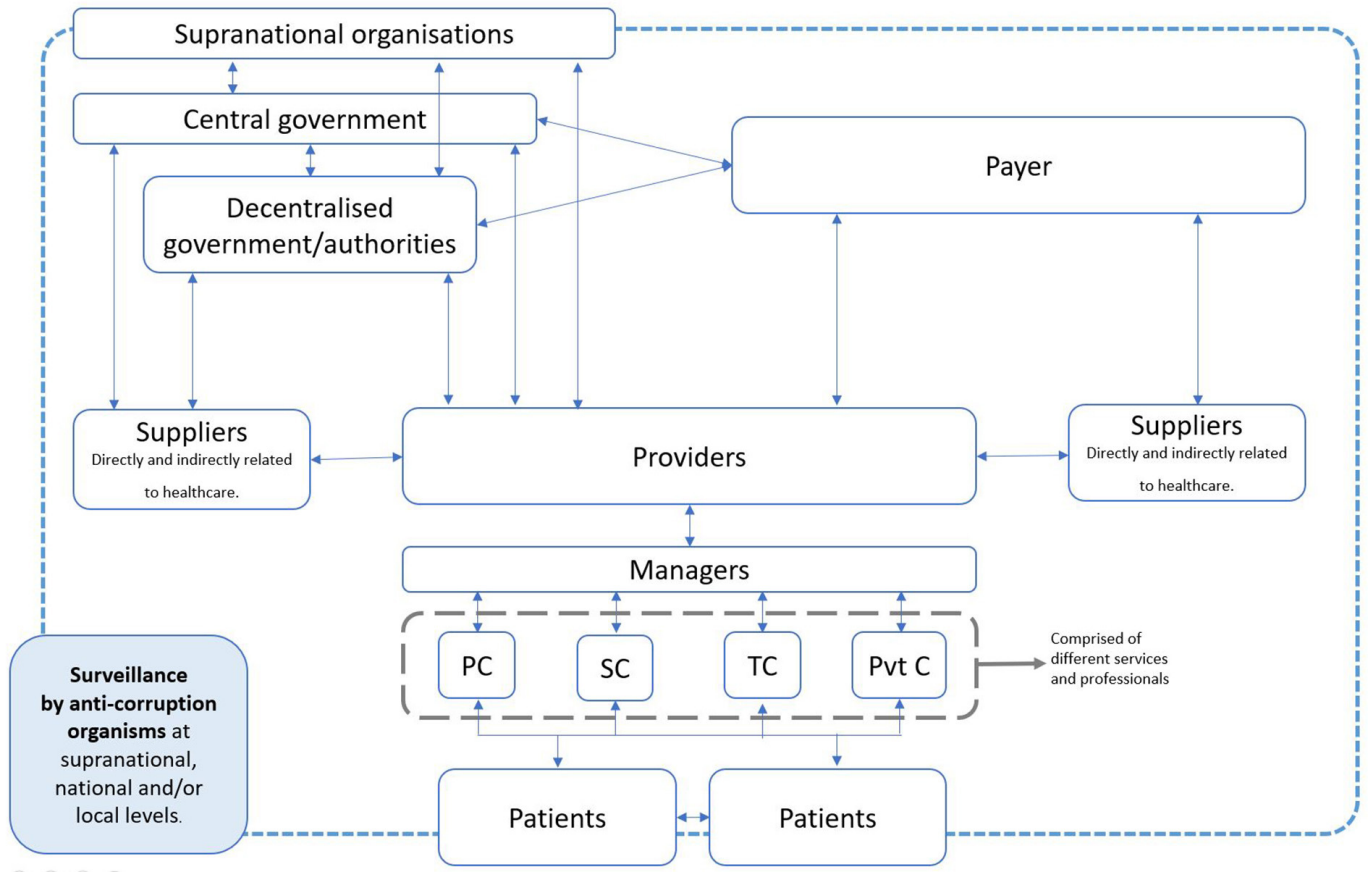
Interactions of actors of a health system. PC, primary care; SC, secondary care; TC, tertiary care; Pvt C, private care. Source: Gonzalez-Aquines et al. (10).

A health system (HS) aims to improve the population's health, achieve responsiveness for improvement of the non-health dimensions of the population, and provide adequate financing and financial risk protection for households (5). In order to achieve its goals, the HS needs to work interdependently with other sectors, leading to a high number of actors involved in several interactions between them (Figure 1). This multiple-sector and multiple-actors characteristic of the HS represents the main vulnerability for corruption in HSs.

Alongside the presence of multiple actors, there is an asymmetric distribution of information, also known as the principal-agent problem, referring to the situation where few (agents) in the health system have the information needed to make the best decision, taking advantage of those without the information (principal) (6). An example during the COVID-19 pandemic can be drawn from the UK when manufacturers (*agents*) of non-health-related industries (e.g., car industry) without the appropriate expertise to produce ventilators obtained funding from governments (*principal*) to increase the availability of this medical device. Although the production increased, the ventilators produced were still not enough to cover the demand during the peaks of the pandemic, and by July 2020, the companies involved returned to their original manufacturing process (7).

## Types of health corruption

One of the main challenges to addressing health corruption is that it can occur at different levels and take multiple faces. As shown in Figure 1, multiple actors might get involved in corrupted activities; however, how they interact might be completely different. While the examples provided before were related to procurement, other types of health corruption exist.

The European Commission provides a simple classification of health corruption based on the Study on Health Corruption in the Healthcare Sector (8):

1. Bribery in medical service delivery,
2. procurement corruption,
3. improper marketing relations,
4. misuse of (high) level positions,
5. undue reimbursement claims, and
6. fraud and embezzlement of medicines and medical devices.

Procurement corruption is the most common type of health corruption due to the significant money transfers involved, although this might vary among countries depending on their historical context. For instance, in post-communist countries, like Slovakia and Poland, there were cases of misuse of (high) level positions, which might have a historical context due to the small and close friendship networks created in the political sphere prevailing even after the communist regime (9).

Increasing the awareness of the different actors and types of health corruption could be the starting point to identify and address it promptly. However, it is fundamental that countries establish effective justice systems as the rule of law measured by the WDI is associated with a reduced perception of corruption (10). Similarly, countries with effective justice systems are more likely to have autonomous institutions (i.e., watchdogs) able to report corruption in the health sector as part of a standard procedure, demanding transparency and accountability to governments. For instance, in the UK, the National Audit Office investigated government procurement during the COVID-19 pandemic, emphasizing the need to follow the required standards to maintain public trust in the government (11).

In addition to effective justice systems, countries must guarantee mechanisms to implement collaborations between the HS actors to ensure a whole-of-government and a whole-of-society approach when tackling corruption. Therefore, we believe that governments and societies could benefit from implementing anticipatory governance practices to ensure corrupt practices are identified and solved timely.

## Anticipatory governance for health corruption

Anticipatory governance allows foreseeing a particular event's consequences (12), such as health corruption during a public health crisis. This type of governance contrasts with good governance, defined as the process involving governments and stakeholders to improve the population's health (13). While governance culminates with creating policies to achieve the HS goals through a linear formulation of policies (e.g., banning tobacco from restaurants after evidence of its harmful impact on health is collected), anticipatory governance practices are developed to prevent harmful practices from occurring and address them on time. Moreover, anticipatory governance requires a whole-of-society approach to increase transparency and accountability; thus, non-governmental organisms and societies are included when implementing anticipatory governance practices.

**Figure 2:**
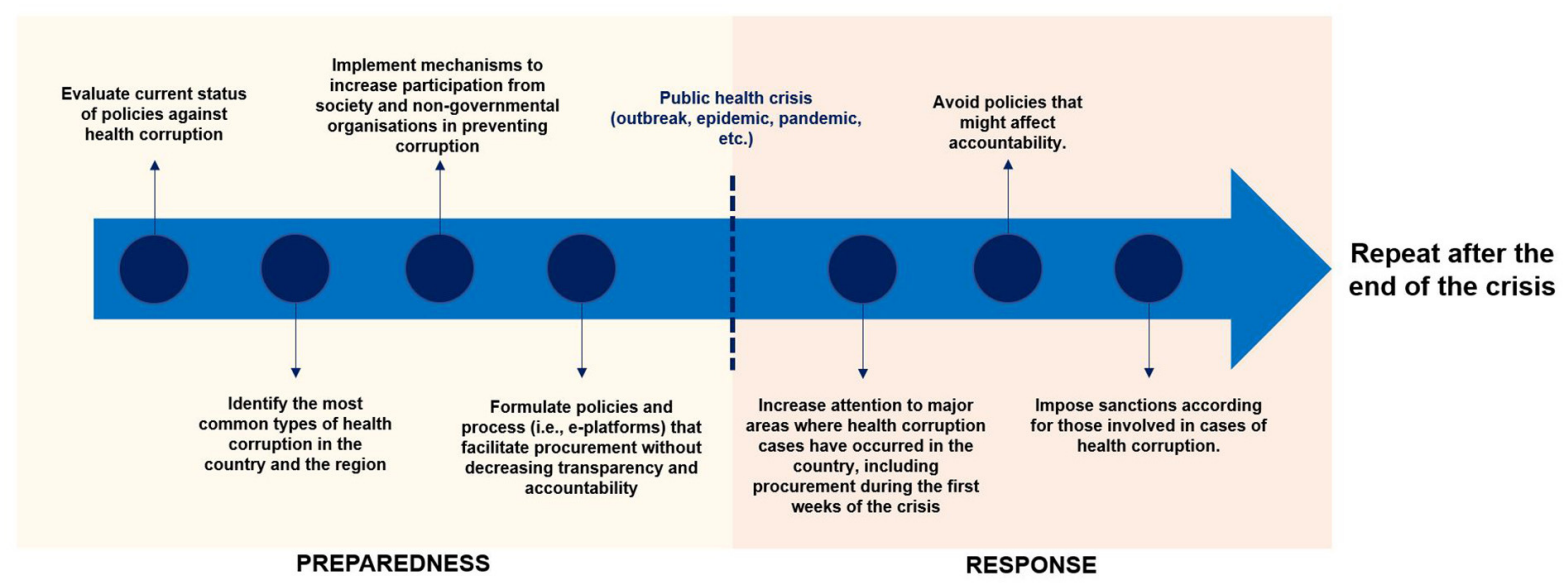
Preparedness and response for health corruption during a public health crisis.

Lessons from the COVID-19 pandemic and previous public health crises have made evident that corruption comes alongside shocks (14). Therefore, HSs should implement adequate preparedness and response to tackle health corruption during future public health crises. Figure 2 presents the authors' proposed strategy to implement anticipatory governance for future public health crises. We highlight that this should be contextualized to each country.

During the preparedness phase, countries should identify the main weaknesses to address corruption in the health sector; for instance, by evaluating the current policies to detect corrupt practices at all levels of health care, from informal payments to healthcare professionals to misuse of high-level positions. Moreover, efficient reporting mechanisms of corrupt practices should be in place while ensuring protection for whistle-blowers. For instance, in the European Union (EU), only nine out of the 27 EU members have adopted the EU Directive on Whistleblowing that entered into force in December 2019 to protect persons reporting breaches of Union law (15, 16). Finally, countries should implement innovative procurement models that do not impose risks to transparency and accountability. An example of this achievable can be drawn from Ukraine by implementing an e-procurement platform in which all emergency contacts must be published within 24 h of being granted (17).

Particular attention should be placed on the response phase at the beginning of the public health crisis, as most corruption cases usually occur at this stage. For instance, the UK National Audit Office revealed that the contracts to acquire PPE at the beginning of the pandemic from March to July 2020 were granted to providers linked to high-level officials (3). Similarly, the novelty and uncertainty of the crisis are used by fraudsters to take advantage of governments and societies looking for crucial equipment needed to protect the population's health. An example of this was observed in the Netherlands and Germany, where German health authorities contacted a Dutch company that claimed to be a face mask provider. The buyers sent a money transfer of 1.5 EUR mill., but a day before the delivery date, the company claimed not to have received the money; the INTERPOL's Financial Crime unit later found that the Dutch company existed, but it was cloned by fraudsters taking advantage of the lack of PPE supply (18). Finally, governments should avoid implementing policies that limit the transparency and accountability of procurements during the crisis, like in the UK, where VIP providers did not go through routine scrutiny, or in Poland, where the government approved a law to wave any penalty for those involved in decisions made during the pandemic, even if the decision was related to corruption (19, 20). Instead, governments must guarantee transparency and accountability and impose clear penalties on those engaging in corrupt practices.

## Conclusion

Health corruption weakens the countries' preparedness and response to the public health crisis, leading to negative consequences on the population's health. In order to make appropriate and efficient use of the resources, governments must address health corruption. Anticipatory governance provides an innovative solution by foreseeing the consequences of issues. By adapting anticipatory governance practices involving key stakeholders, countries would be able to avoid losing money in corrupted activities and save lives by increasing the use of resources to strengthen their health systems. There is no doubt that we need to be prepared for future pandemics, but more importantly, we also need to be ready to tackle corruption during future public health crises.

## Data availability statement

The original contributions presented in the study are included in the article/supplementary material, further inquiries can be directed to the corresponding author.

## Author contributions

All authors listed have made a substantial, direct, and intellectual contribution to the work and approved it for publication.

## Conflict of interest

The authors declare that the research was conducted in the absence of any commercial or financial relationships that could be construed as a potential conflict of interest.

## Publisher's note

All claims expressed in this article are solely those of the authors and do not necessarily represent those of their affiliated organizations, or those of the publisher, the editors and the reviewers. Any product that may be evaluated in this article, or claim that may be made by its manufacturer, is not guaranteed or endorsed by the publisher.
